# Characterization of the complete mitochondrial genome of the orange swift, *Triodia sylvina*

**DOI:** 10.1080/23802359.2018.1467223

**Published:** 2018-04-27

**Authors:** Chao Li, Lili Li, Zengbin Lu, Yixin Sun, Li Liu, Song Dong, Xingyuan Men

**Affiliations:** a College of Marine Life Sciences, Ocean University of China, Qingdao, China;; b Institute of Plant Protection, Shandong Academy of Agricultural Sciences, Jinan, China;; c Maize Research Institute, Shandong Academy of Agricultural Science, Jinan, China;; d College of Plant Protection, Shandong Agricultural University, Taian, China

**Keywords:** *Triodia sylvina*, mitochondrial genome, assembly, phylogeny

## Abstract

In this study, the complete mitochondrial genome of orange swift, *Triodia sylvina*, was recovered through Illumina sequencing data. This complete mitochondrial genome of *T. sylvina* is 15,040 bp in length and has a base composition of A (41.0%), T (41.2%), C (10.4%), G (7.5%), demonstrating an extreme bias of high AT content (82.2%). The mitochondrial genome contains a typically conserved structure among moth mitogenomes, encoding 13 protein-coding genes (PCGs), 22 transfer RNA (tRNA) genes, two ribosomal RNA (12S rRNA and 16S rRNA) genes and a control region (D-loop region). Seven PCGs were located on the H-strand, others were located on the L-strand. *ATP8* gene and *ATP6* gene were overlapped by 6 bp. *ND4* gene and *ND4L* gene were overlapped by 9 bp. The whole mt genome of *T. sylvina* and other moth and butterfly mitogenomes (100 species, in total) were used for phylogenetic analysis. The result indicated *T. sylvina* represents a distinct genus, which separated far from genus *Hepialus*.

*Triodia sylvina* is a species of moth belonging to the family Hepialidae. It has been previously placed in the genus *Hepialus*, and is distributed throughout Europe. *T. sylvina* is attracted by artificial light and the larva feeds on the roots of various plants including bracken, dandelion, dock, hop and viper’s bugloss. Until now, the organelle genome information of *T. sylvina* is still limited. In this study, the complete mitochondrial genome of *T. sylvina* was recovered through Illumina sequencing data. This complete mitochondrial genome can be subsequently used for testing the origin of the W chromosome and provide valuable insight into phylogeny relationship among Lepidoptera species (Fraïsse et al. 2017).

The female sample of *T. sylvina* was collected from Hellerup, Denmark (55°74′N, 12°56′W). Genomic DNA was extracted from two legs using QLAGEN DNEasy Extraction Kit following the manufactures instructions. The isolated DNA was stored in the sequencing company (BGI Tech, Shenzhen, China). Purified DNA was fragmented and used to construct the sequencing libraries following the instructions of NEBNext^®^ Ultra™ II DNA Library Prep Kit (NEB, BJ, CN). Whole genomic sequencing was performed by the Illumina HiSeq 2500 Sequencing Platform (Illumina, San Diego, CA). Adapters and low-quality reads were removed using the NGS QC Toolkit (Patel and Jain 2012). Then assembly as implemented by SPAdes 3.9.0 (Bankevich et al. 2012). Circularization of this mt genome was confirmed using MITObim V1.9 (Hahn et al. 2013). The complete sequence was primarily annotated by ORF prediction in Unipro UGENE (Okonechnikov et al. 2012) combined with manual correction. All tRNAs were confirmed using the tRNAscan-SE search server (Lowe and Eddy 1997). Other protein-coding genes (PCGs) were verified by BLAST search on the NCBI website (http://blast.ncbi.nlm.nih.gov/), and manual correction for start and stop codons were conducted. This complete mitochondrial genome sequence together with gene annotations was submitted to GenBank under the accession numbers of MH105794.

The complete mitochondrial genome of *T. sylvina* was 15,040 bp in length and has a base composition of A (41.0%), T (41.2%), C (10.4%), G (7.5%), demonstrating an extreme bias of high AT content (82.2%). The mitochondrial genome contains a typically conserved structure among moth mitogenomes, encoding 13 PCGs, 22 transfer RNA (tRNA) genes, two ribosomal RNA (12S rRNA and 16S rRNA) genes and a control region (D-loop region). Seven PCGs were located on the H-strand, others were located on the L-strand. *ATP8* gene and *ATP6* gene were overlapped by 6 bp. *ND4* gene and *ND4L* gene were overlapped by 9 bp.

For phylogenetic analysis assessing the relationship of this mitogenome, we selected other 100 mt genomes from moths (22 taxa) and butterflies (78 taxa) to construct genome-wide alignment. The genome-wide alignment of all cp genomes was done by HomBlocks (Bi et al. 2018), resulting in 12,488 positions in total, including almost all PCGs and rRNA genes. The whole genome alignment was analysed by PhyloBayes ver. 3.3 (Lartillot et al. 2009) under the GTR + G model that accounts for across-site heterogeneities. Four independent MCMC analyses were run for 10,000 cycles in PhyloBayes. Convergence was checked based on time-series plots of the likelihood scores using Tracer (http://tree.bio.ed.ac.uk/software/tracer/). The first 5000 cycles were discarded as burn-in, and the remaining trees were summarized to obtain Bayesian posterior probabilities (BPPs). The resulting tree was represented and edited using FigTree v1.4.1. As shown in Figure 1, the phylogenetic positions of these 101 mt genomes were successfully resolved with high BPPs supports across almost all nodes. *T. sylvina* was placed in a single branch, which separated far from genus *Hepialus*, presenting the genus *Triodia.*

**Figure 1.**
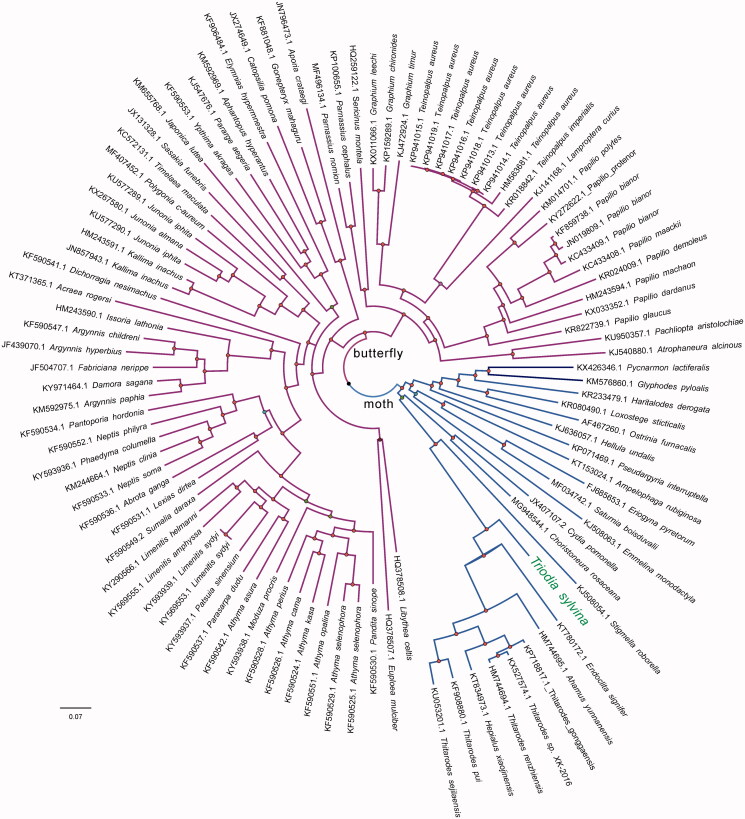
Phylogenetic tree yielded by Bayesian analysis of 101 mt genomes. Bayesian consensus tree is shown with support indicated by numbers at branches, representing Bayesian posterior probabilities (BPPs). Fully phylogenetical resolved nodes are labelled by red points.
